# A rapid method for determination of rosuvastatin in blood plasma with supported liquid extraction

**DOI:** 10.1016/j.jmsacl.2025.04.003

**Published:** 2025-04-10

**Authors:** Tjaša Dermota, Mojca Božič Mijovski, Jurij Trontelj

**Affiliations:** a University Medical Centre Ljubljana, Slovenia; b Faculty of Pharmacy, University of Ljubljana, Slovenia

**Keywords:** SLE, Supported Liquid Extraction, LLE, Rosuvastatin, LC-MS/MS

## Abstract

**Introduction:**

Accurate measurement of rosuvastatin in plasma is critical for effective patient management and treatment monitoring following myocardial infarction (MI). Expensive solid-phase extraction (SPE) and time-consuming liquid–liquid extraction (LLE) have been established for quantifying rosuvastatin. Supported liquid extraction (SLE) could offer a rapid, cost-effective alternative.

**Objectives:**

This study aimed to develop and validate a rapid, cost-effective, accurate, and precise method for quantifying rosuvastatin in high-dose plasma samples from patients following MI.

**Methods:**

Rosuvastatin was extracted from EDTA plasma using SLE and quantified with LC-MS/MS with positive electrospray ionization. The method was validated according to ICH M10 guidelines, focusing on selectivity, matrix effect, accuracy, precision, linearity, and carryover. Rosuvastatin-D6 was used as an internal standard. Additionally, thirty plasma samples from patients on high-dose rosuvastatin therapy (20 or 40 mg/day) following MI were analyzed by both LLE and SLE methods and compared.

**Results:**

The method was successfully validated, demonstrating linearity across a range of 0.1 ng/mL to 50 ng/mL. Compared to the LLE method, SLE achieved superior extraction recovery (96.3 % vs. 60 %) and precision (RSD: 11.9 % vs. 13.6 %) at 0.3 ng/mL rosuvastatin, with a lower absolute matrix effect (12.7 % vs. −36.7 %). Accuracy was comparable (109.3 % vs. 92.8 %). Although SLE involves higher initial costs, it significantly enhances throughput, reduces solvent usage, and minimizes contamination and equipment wear.

**Conclusion:**

This study validates SLE as a superior method for quantifying rosuvastatin in plasma, outperforming LLE in recovery, reproducibility, and automation. SLE offers greater accuracy and reliability, making it ideal for high-throughput applications.

## Highlights

• SLE outperforms LLE in most validation parameters.
• SLE demonstrates linearity across a range of 0.1 – 50 ng/mL.
• SLE has better extraction recovery and reproducibility than LLE.
• SLE offers better cleanup with lower matrix effect and cleaner extracts.
• SLE consistently showed 14.6% higher rosuvastatin responses in clinical samples.

## Introduction

Atherosclerotic cardiovascular disease (ASCVD) accounts for as much as 45 % of premature deaths.

with about 11 % attributed to acute myocardial infarction (MI). The primary risk factors for ASCVD include lipoprotein particles containing apolipoprotein B (ApoB), with low-density lipoprotein (LDL) being the most common, along with high blood pressure, smoking, obesity, and diabetes [1], [2].

Rosuvastatin is a lipid-lowering synthetic drug and can be prescribed in various doses, ranging from 5 to 40 mg per day. The oral bioavailability of rosuvastatin after a single dose is 20 %, while absorption is approximately 50 % [3]. The pharmacokinetics of rosuvastatin is affected not only by the dose but also by genetic polymorphisms, especially in transport proteins ABCG2 and SLCO1B1, race, co-administration of other drugs, and the presence of other non-cardiovascular diseases. As a result, the concentration can vary greatly between individuals, affecting its efficacy and toxicity [4], [5], [6].

Since rosuvastatin is excreted largely unchanged, with about 90 % not undergoing metabolism [7], this paper will focus solely on the quantification of the unmetabolized molecule. Several validated methods have already been established for quantifying rosuvastatin concentrations in plasma. The majority of these methods use liquid–liquid extraction (LLE), which allows for a lower limit of quantification (LLOQ) as low as 0.02 ng/mL [8], [9], [10], [11], [12], [13], [14], [15], [16], [17], while others use solid-phase extraction (SPE) [18], [19]. However, to the best of our knowledge, none of them have utilized supported liquid extraction (SLE) for sample preparation.

LLE is a widely used extraction technique that separates compounds based on their solubility differences between two immiscible liquid phases, typically an aqueous phase and an organic solvent. Following mixing, phase separation is facilitated by centrifugation, with analytes preferentially transferring into the organic layer. Similarly, SLE leverages differential solubility but utilizes porous solid support, such as diatomaceous earth, to adsorb aqueous samples. Upon applying an organic solvent, the analytes efficiently partition into the organic phase, enabling effective separation from more polar matrix components which remain adsorbed. [20] The SLE method is expected to have better recovery and reproducibility compared to LLE [21]. Additionally, the SLE process is much easier to automate [22]. Unlike LLE, there is no need for vigorous shaking or careful pipetting to separate the layers. Furthermore, since the SLE method is cartridge-based, it enables the simultaneous processing of multiple samples, which has the potential to significantly increase throughput, and simultaneously reduces the consumption of organic solvents [22]. In comparison to SPE, SLE is a faster and more cost-effective method since cartridge preparation and wash steps are not required [23], [24], [25].

The primary objective of this study was to develop a rapid, accurate, and precise method for the quantification of rosuvastatin in plasma samples from patients on high-dose rosuvastatin. To assess feasibility as well as performance, two sample preparation methods were compared: the novel SLE and classical LLE as a reference. We also aimed to validate the SLE method with LC-MS/MS detection for quantifying rosuvastatin concentration in the plasma of patients receiving high-dose rosuvastatin therapy (20 or 40 mg/day) after a MI to assess its ability to monitor patient adherence to therapy and to evaluate how patients respond to rosuvastatin treatment, as MI can influence the pharmacokinetic profile of the drug.

## Materials and methods

### Chemicals and Instrumentation

**Fig. 1:**
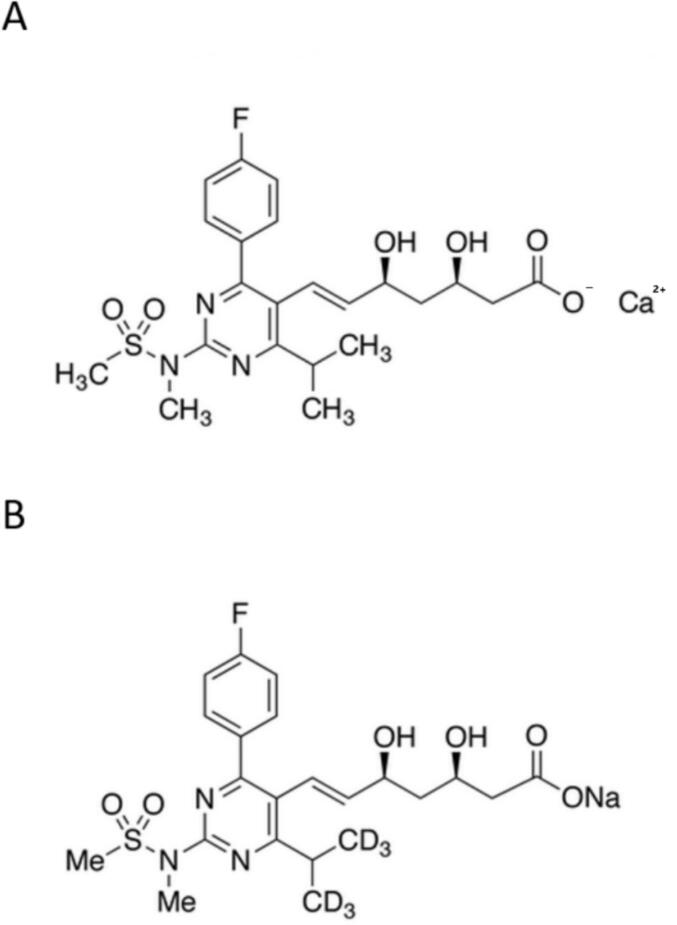
The chemical structures of rosuvastatin, Sequoia Research Products Ltd. (A) and Rosuvastatin-D6 Sodium, Toronto Research Chemicals (B).

Rosuvastatin calcium (95 % purity) for quality control and standard solutions was provided by Sequoia Research Products Ltd. As the internal standard (IS), rosuvastatin D6 sodium was obtained from Toronto Research Chemicals (Fig. 1). All solvents used for the mobile phase were MS-grade. Acetic acid, 99.9 % acetonitrile, methanol, formic acid, isopropanol (IPA) and *tert*-butyl methyl ether (TBME) from Honeywell and ammonium acetate from Merck were used. Ultrapure water purified with the Milli-Q water purification system (Millipore) was utilized. University Medical Center Ljubljana (Slovenia) provided pooled human plasma with EDTA as an anticoagulant. For SLE extraction, Novum SLE 3 cc tubes from Phenomenex were used.

The analytes extracted from biological samples were analyzed using an Agilent 1290 Infinity II ultra-high-performance liquid chromatograph (UHPLC) coupled to a triple quadrupole/linear ion trap mass detector, the Sciex QTRAP 5500+.

### Preparation of standard Solutions, calibration and quality control samples

Rosuvastatin and rosuvastatin-D6 were separately and accurately weighed and dissolved in methanol to achieve a concentration of 1 mg/mL. Subsequently, two secondary stock solutions of rosuvastatin (1000 ng/mL and 100 ng/mL) were prepared. Both were stored at 4 °C. They were further diluted with 25 % methanol to obtain working solutions with concentrations of 1, 2, 3, 5, 10, 20, 50, 100, 150, 200, 400 and 500 ng/mL. The IS was diluted with 25 % methanol to reach a final concentration of 10 ng/mL, aliquoted, and stored at −20 °C.

Calibration and quality control (QC) samples were prepared by spiking blank plasma (900 μL) with working solutions (100 μL) of rosuvastatin, resulting in concentrations of 0.1, 0.2, 0.5, 1, 2, 5, 10, 20, and 50 ng/mL for the standard curve and 0.3, 15, and 40 ng/mL for the QCs. QCs were prepared from separate weighings. Aliquots (100 μL) were stored at −20 °C until needed.

### Sample extraction

For both extraction methods, aliquots of QC and calibration samples were thawed at room temperature. Then, 50 μL of IS (10 ng/mL) was added to the previously spiked plasma.

#### SLE

To 150 μL of spiked plasma, 200 μL of ammonium acetate (100 mM in water, pH 3.5) was added and briefly vortexed. Subsequently, 325 μL of the sample was loaded onto the solid support and soaked into the sorbent using a vacuum. After 15 min of incubation, 1000 μL of TBME + 10 % IPA was applied. The organic phase was eluted by gravity into a clean tube and evaporated to dryness under a stream of nitrogen in a water bath at 40 °C. The residue was reconstituted with 100 μL of reconstitution solvent (0.1 % formic acid in 70 % MeOH diluted with Milli-Q water).

#### LLE

To 150 μL of spiked plasma, 100 μL of ammonium acetate (100 mM in water, pH 3.5) and 850 μL of organic phase (TMBE + 10 % IPA) were added. Samples were thoroughly vortexed and shaken for 1 h at 250 rpm on an orbital shaker (Vibromix EVT403, Tehtnica, Železniki, Slovenia) at room temperature, followed by a 10 min centrifugation at 16,000 × *g*. Then, 750 μL of the organic phase was transferred to a new tube, evaporated to dryness, and reconstituted as previously described above for SLE. Since suspended particles were observed after reconstitution, a centrifugation step of 30 min at 3,000 × *g* was added.

### LC-MS/MS Conditions

For HPLC, the mobile phase was composed of Solvent A: 0.025 % acetic acid in Milli-Q water and Solvent B: 99.9 % acetonitrile, delivered at a rate of 0.5 mL/min. The change over time in the percentage of solvent B is shown in Table 1. The column used was Agilent Poroshell 120 EC-C18 (50 x 2.1 mm with 1.9 µm particles) and was maintained at 40 °C. The total run time was 4 min, with an injection volume of 0.5 μL, and the autosampler temperature set to 10 °C.

**Table 1:** The mobile phase gradient used for rosuvastatin elution.

Time [min]	Percent of solvent B [%]
0	20
1.0	65
1.2	70
1.4	90
3.2	90
3.4	20

The LC-MS interface operated in positive mode with electrospray ionization, with nebulizer gas (GS1) set at 30 psi, heating gas (GS2) at 58 psi, collision gas at 9 psi, and drying gas at 23 psi. The heater temperature was 650 °C, and the ion spray voltage was set to 5500 V. The samples were analyzed by multiple reaction monitoring (MRM) using the following *m*/*z* transitions: for rosuvastatin, 482.2 to 258.1 (quantifier) at collision energy of 45 eV and cell exit potential (CXP) of 8 V, and *m*/*z* 482.2 to 300.2 (qualifier) at collision energy of 49 eV and CXP of 15 V; for the IS, 488.2 to 306.2 (quantifier) at collision energy of 49 eV and CXP of 15 V, and 488.2 to 264.1 (qualifier) at collision energy of 45 eV and CXP of 14 V. The declustering potential (DP) was 126 V, and the entrance potential was 10 V for all ion transitions.

### Method validation

In this study, our focus was on validating the SLE method and comparing it to the already validated LLE method. The SLE method was validated according to the purposes of our study, namely to reliably quantify rosuvastatin in steady-state samples from MI patients receiving a 40 mg dose. The validation parameters for selectivity, matrix effect, accuracy, precision, linearity, and carryover were based on the ICH M10 bioanalytical method validation guidelines [26].

Selectivity evaluation involved analyzing double-blank samples (samples without analyte or IS) from six different individuals. The expected responses at the retention times of rosuvastatin and the IS should be less than 20 % of the rosuvastatin response at the LLOQ and 5 % of the IS response.

The relative matrix effect (RME) was assessed by analyzing three replicates of low (0.3 ng/mL) and high (40 ng/mL) QC samples, each prepared in blank plasma from six different individuals. The expected coefficient of variation (CV) should not be greater than 15 %. Additionally, the absolute matrix effect (AME) was evaluated by comparing the response of pre-spiked (QC) samples with the response of the reference solution prepared in neat solvent corresponding to the final QC concentrations after preparation. To assess the matrix effect in lipemic and hemolyzed samples, pooled blank plasma samples with each type of interference were spiked in triplicate at low and high QC levels and analyzed.

Equation 1: Relative matrix effect (RME) calculation.

$$\mathrm{RME}\;\left[\%\right]=\frac{\mathrm{Standard}\;deviation}{\mathrm{Average}}\times 100\%$$

Equation 2: Absolute matrix effect calculation (AME). B represents the response of pre-spiked (QC) samples, C represents the response of the reference solution prepared in a neat solvent.

$$\mathrm{AME}\;\left[\%\right]=\;\frac{B}{C}\times 100\%$$

The extraction recovery (ER) was assessed by comparing the peak areas of three replicates of low and high QC samples spiked before sample preparation with the peak areas of blank plasma samples spiked after preparation.

Equation 3: Extraction recovery (ER) calculation. A represents the peak areas of samples spiked before sample preparation, while B is the peak area of a blank plasma sample spiked after preparation.

$$\mathrm{ER}\;\left[\%\right]=\frac{A}{B\;}\times 100\%$$

A calibration curve was prepared on three separate days, including a blank (blank plasma with IS) and a double-blank sample, along with nine different concentrations of rosuvastatin (ranging from 0.1 to 50 ng/mL). The linearity of the calibration curves over three consecutive days was assessed using the lack-of-fit test and visually examined by overlaying the curves on the same graph and comparing their slopes and intercepts to the average values. The LLOQ was determined by analyzing five separate replicates of samples at a concentration of 0.1 ng/mL. The response of the analyte at the LLOQ should be fivefold higher than the response of the double-blank sample.

Between-run accuracy and precision were evaluated by analyzing five replicates of spiked QCs at three different concentration levels (0.3, 15 and 40 ng/mL) on three different days. Within-run accuracy and precision were also assessed by analyzing five replicates at each QC concentration level in individual analytical runs. The expected accuracy was within ± 15 % of the nominal concentration, and the precision (%CV) was not greater than 15 %, except at the LLOQ, where the accuracy and precision should be 80–120 %, and the relative standard deviation (RSD) should be < 20 %.

Equation 4: Accuracy calculation

$$\mathrm{Accuracy}=\frac{\mathrm{Measured}\;value}{\mathrm{True}\;value}\times 100\%$$

Carryover was assessed by analyzing blank samples injected after the sample at the upper limit of quantification (50 ng/mL). The expected response should be lower than 20 % of the rosuvastatin response at the LLOQ and 5 % of the response for the IS.

The stability of rosuvastatin in plasma has already been extensively studied. It has been proven to be stable when stored at −70 °C, as well as at −20 °C, for at least 6 months. It was also stable for three freeze/thaw cycles [18]. Nevertheless, we wanted to confirm the benchtop stability for 2 h to simulate the scenarios that might be encountered in our study.

### The method comparison study on patient samples

Blood samples were collected in tubes containing EDTA at the University Medical Centre Ljubljana after Ethics Committee approval from the National Medical Ethics Committee of the Republic of Slovenia (Approval Nr.: 0120–124/2023/7). Thirty samples from different patients were collected at various predetermined time points following the last rosuvastatin administration. Each blood sample was centrifuged immediately at 2,000 × *g* for 15 min, and plasma was transferred to a clean tube and stored at −70 °C. Before analysis, the samples were thawed at room temperature. Rosuvastatin was extracted using two different methods: SLE and LLE. For comparison, Bland-Altman Plot and regression analysis were used.

## Results and discussion

### Method validation

#### Selectivity, matrix effect, carryover and recovery

**Fig. 2:**
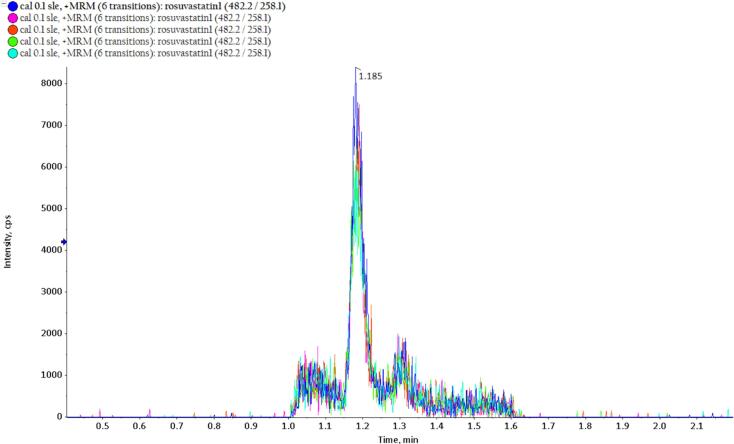
MRM chromatogram of 5 different rosuvastatin samples (LLOQ).

**Fig. 3:**
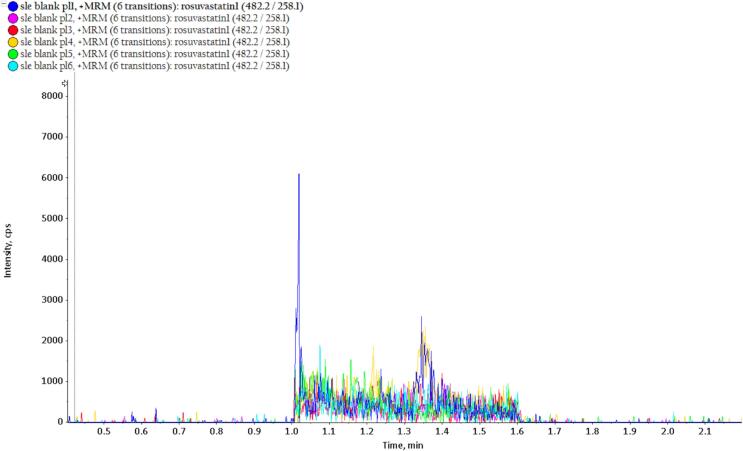
MRM chromatogram of 6 different blank samples.

The MRM chromatograms of the LLOQ and six different blank plasma samples are shown in Fig. 2 and Fig. 3, respectively. At the retention time of rosuvastatin, approximately 1.2 min, no significant interference peaks were observed in the blank samples. Additionally, analyte peak areas of the blank samples were lower than 15 %, and for IS, less than 5 % of the peak areas in the sample at LLOQ, indicating acceptable selectivity of the method.

The CV in determining the RME in six individual plasmas was 5.2 % for rosuvastatin and 5.9 % for IS at the low-level QC, while at the high-level QC, the variation was slightly lower at 4.3 % (rosuvastatin) and 4.2 % (IS), suggesting a relatively low matrix effect and indicating consistent and reliable measurements across different plasma samples.

The AME at 0.3 ng/mL and 40 ng/mL rosuvastatinwas 12.7 % and −6.2 % for rosuvastatin, and 11.1 % and −0.89 % for the IS, respectively. The observed matrix effects for both rosuvastatin and the IS align with the acceptable range of ± 15 %.

The mean ER of rosuvastatin at two QC levels and the IS were 96 %, 83 %, and 76 %, respectively. These values indicate the efficiency of the extraction process in recovering the analyte from the plasma matrix.

The matrix effect in lipemic plasma was −3.7 % at the QCL level and 11.7 % at the QCH, while in hemolyzed plasma, it was −5.0 % for QCL and −4.5 % for QCH, indicating the absence of a significant impact from the tested interferences.

The response of a double-blank sample injected directly after the highest calibration standard was 5.3 % of the LLOQ response for the analyte and 0.37 % for the IS.

The study confirms the method's suitability for analyzing samples with lipemic or hemolyzed characteristics, and the low responses in the double-blank underscore the precision and lack of interference in the analytical system. These findings support the reliability and robustness of the method, meeting the criteria for both matrix effects and the response of double-blanks.

#### The calibration curve and range (LLOQ)

**Fig. 4:**
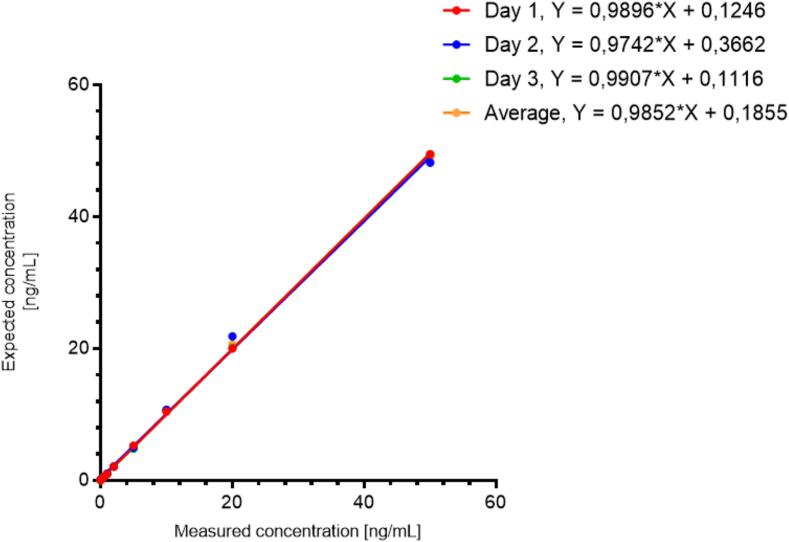
Calibration curves on three different days (red, blue and green line) and their average values (orange line) with linear regression equations. (For interpretation of the references to colour in this figure legend, the reader is referred to the web version of this article.)

The calibration curve displayed acceptable linearity, and the determination coefficient (R^2^) exceeded 0.999, ensuring reliable assay performance across a range from 0.1 to 50 ng/mL. The slopes and intercepts of all three calibration curves were similar, as shown in Fig. 4, indicating no significant differences between the curves obtained on different days. The lack-of-fit test showed that the linear regression model adequately describes the calibration (F_calc_. = 1.639, F_critical_ = 2.577). The LLOQ was determined over three different days at 0.1 ng/mL, with an RSD of 11.9 %, indicating acceptable precision and an accuracy of 94.1 %. The response at the LLOQ was approximately 80 times higher than the response in the double-blank sample. This large difference indicates a robust signal-to-noise ratio, demonstrating the ability to detect low concentrations of the analyte against a background of blank samples.

#### Accuracy and precision

Accuracy and precision were assessed at three concentration points (0.3, 15 and 40 ng/mL). Inter-day repeatability was evaluated over three different days, and intra-day repeatability was conducted with five replicates. The results are presented in Table 2. At all three concentration levels, accuracy and precision met the acceptance criteria.

**Table 2:** Inter-day and Intra-day variability and accuracy of rosuvastatin quantification.

Nominal conc. [ng/mL]	Intra-day			Inter-day		
	Mean (ng/mL)	RSD	Accuracy (%)	Mean (ng/mL)	RSD	Accuracy (%)
0.3	0.3	13.2	99.3	0.3	11.9	109.3
15	15.0	7.8	100.3	15.7	3.2	104.8
40	40.4	3.6	101.1	39.5	2.9	98.7

RSD-relative standard deviation

#### Stability

The bench-top stability of two hours was confirmed at low-level QC and high-level QC. The mean concentrations were 0.34 and 41.07 ng/mL, with relative errors (RE) of 14.8 % and 2.7 %, respectively.

### Application to a method comparison study

In the second phase of the study, the results of the validated method were compared with an already established method that utilizes liquid extraction for sample preparation (Table 3). Using the SLE method, we were able to reliably determine the analyte concentration at 0.1 ng/mL, whereas with the LLE method, we had to set the LLOQ slightly higher (0.3 ng/mL) due to poor accuracy (73.5 %) and precision (RSD 73 %) at lower concentrations. The SLE method showed higher responses in QC samples, especially at lower concentrations (109.3 % vs. 92.8 %), which may be due to higher extraction recovery at lower levels (96.3 % vs. 60.0 %) and lower matrix effects (12.7 % vs. −36.7 %). Furthermore, there was better precision across all concentrations, especially at 15 ng/mL (3.2 % vs. 7.3 %). Although the differences are minor, the results indicate that SLE provides more consistent and reliable measurements.

**Table 3:** Comparison of validation parameters of SLE and LLE.

Validation parameter		SLE	LLE*
Linearity (ng/mL)		0.1–50	0.3–50
Accuracy (%)	0.3 ng/mL	109.3	92.8
	15 ng/mL	104.8	98.5
	40 ng/mL	98.7	100.8
Precision-RSD (%)	0.3 ng/mL	11.9	13.6
	15 ng/mL	3.2	7.3
	40 ng/mL	2.9	3.3
ER (%)	0.3 ng/mL	96.3	60.0
	40 ng/mL	82.3	83
AME (%)	0.3 ng/mL	12.7	−36.7
	40 ng/mL	−6.2	−6.38
RME (%)	0.3 ng/mL	5.2	6.09
	40 ng/mL	4.3	2.16

* The data on LLE were generated in our laboratory.

AME-absolute matrix effect, ER-extraction recovery, RME-relative matrix effect, RSD-relative standard deviation

The analysis costs per sample are higher with SLE, as, in addition to the reagents, cartridges are also required. In approximately 50 % of the samples analyzed with LLE, a precipitate was observed, whereas no precipitate was visible with SLE. Consequently, HPLC components may be more adversely affected by LLE, thereby increasing analysis costs arising from the shorter lifetime of chromatographic consumables and unpredictable hardware problems. Moreover, LLE is more demanding for analysts, consumes more time, and presents greater challenges for automation.

Table 4 displays the summary data of rosuvastatin concentrations in patient plasma for both methods, with the assessment of normality conducted through the Shapiro-Wilk test. The LLE method produced lower results, with a median concentration of 7.4 ng/mL (Min-Max 2.4–37.9 ng/mL), compared to the SLE method, which had a median of 9.1 ng/mL (2.18–35.1 ng/mL). Neither of the methods showed a normal distribution of the data.

**Table 4:** Summary data of rosuvastatin concentrations for SLE and LLE patients’ sample preparation.

	SLE	LLE
N	30	30
Min-Max [ng/mL]	2.39–37.9	2.18–35.1
Mean ± SD [ng/mL]	14.3 ± 11.5	12.5 ± 10.4
Median (IQR) [ng/mL]	9.1 (11.7)	7.4 (10.7)
P (normality)	<0.0001	<0.0001

IQR-interquartile range, N – number of samples, SD-standard deviation

**Fig. 5:**
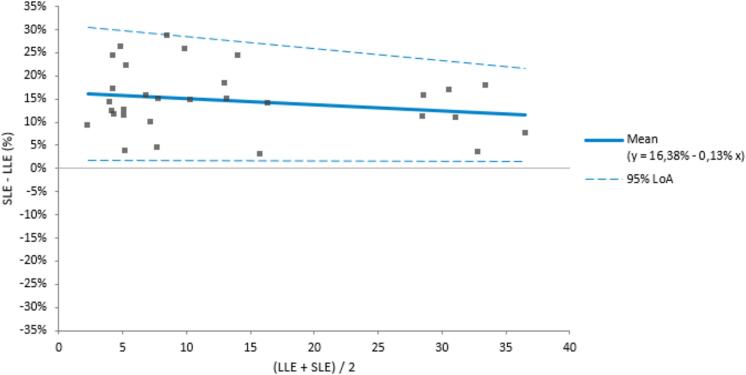
Bland-Altman analysis for the comparison of SLE and LLE methods with N = 30.

The Bland-Altman analysis (Fig. 5) on 30 samples was performed to assess the relative differences between the methods using a linear mean function (D = α + βZ) and a linear standard deviation (SD) function as described in Equation 5. Both p-values were found to be higher than 0.05, indicating that there is no significant variability or trends in the relative differences and SD. The mean difference between the methods was calculated to be 14.64 % with a 95 % CI of 12.08–17.21 %, a SE of 1.25 % and an SD of 6.9 %.

Equation 5: linear SD function

$$\sqrt{\pi^{2}}*\left|R\right|=\alpha +\beta Z$$

The concentrations obtained through LLE consistently exhibited lower values in comparison to those obtained through SLE. The Passing-Bablok regression analysis indicated a linear relationship between the methods (SLE = 0.1192 + 1.144*LLE) with an intercept 95 % CI of −0.1942 to 0.6199 and a slope 95 % CI of 1.079 to 1.189. The variation in results may stem from differences in extraction efficiency, matrix effects, specificity, and method sensitivity. Consistent with findings in other studies comparing these methods [27], [28], our results suggest that SLE's higher recovery is a probable key factor contributing to the elevated outcomes in our case. Considering the wide rosuvastatin dosage range (5–40 mg daily) and high pharmacokinetic variability, the observed bias between both methods is not likely to lead to any significant clinical decisions, such as the adjustment of dosage or switching of medications. However, a method with better recovery, higher sensitivity, and lower matrix effects may offer important advantages for the accuracy of pharmacokinetic data obtained in specific populations where small differences may allow for a better understanding of rosuvastatin disposition in individual patients.

In summary, our validated SLE method presents an important upgrade over the established LLE method, as it offers several key benefits, such as better potential for automation, a lower limit of quantitation, improved extraction recovery, reduced matrix effects, enhanced repeatability, and, most importantly, cleaner extracts, which significantly improves method robustness and reduces instrument contamination and downtime. These advantages may justify the higher up-front costs associated with SLE over the long term.

## Conclusion

This study validates SLE as a robust alternative to LLE for quantifying rosuvastatin in plasma. SLE exhibits superior recovery, reproducibility, and automation potential. The method validation confirms its reliability and sensitivity, making it a promising choice for high-throughput applications. The method comparison study with patient samples suggests that LLE consistently underestimates concentrations compared to SLE, providing valuable insights for researchers and clinicians. Overall, SLE is an efficient and validated method for rosuvastatin quantification.

## Ethics Statement

Blood samples were collected at the University Medical Centre Ljubljana after Ethics Committee approval from the National Medical Ethics Committee of the Republic of Slovenia (Approval Nr.: 0120–124/2023/7). The study was carried out in accordance with the The Code of Ethics of the World Medical Association (Declaration of Helsinki). All proper approvals have been collected. The research conforms to all ethical requirements in effect in the jurisdiction in which the research was conducted and the privacy rights of human subjects have been maintained.

## Funding support

The study was supported by the Slovenian Research Agency [Grant Nr.: P3-0308 and P1-0189].

## CRediT authorship contribution statement

**Dermota Tjaša:** Writing – original draft, Validation, Methodology, Investigation, Formal analysis. **Božič Mijovski Mojca:** Writing – review & editing, Supervision, Resources. **Trontelj Jurij:** Writing – review & editing, Supervision, Project administration, Data curation, Conceptualization.

## Declaration of competing interest

The authors declare that they have no known competing financial interests or personal relationships that could have appeared to influence the work reported in this paper.
